# Quantification and Chemical Characterization of Plastic Additives and Small Microplastics (<100 μm) in Highway Road Dust

**DOI:** 10.3390/toxics11110936

**Published:** 2023-11-17

**Authors:** Beatrice Rosso, Barbara Bravo, Elena Gregoris, Carlo Barbante, Andrea Gambaro, Fabiana Corami

**Affiliations:** 1 Department of Environmental Sciences, Informatics and Statistics, Ca’ Foscari University of Venice, Via Torino 155, 30172 Venice, Italy; beatrice.rosso@unive.it (B.R.); elena.gregoris@cnr.it (E.G.); carlo.barbante@cnr.it (C.B.); gambaro@unive.it (A.G.); 2 Thermo Fisher Scientific, Str. Rivoltana, Km 4, 20090 Rodano, Italy; barbara.bravo@thermofisher.com; 3 Institute of Polar Sciences, CNR-ISP, Campus Scientifico, Ca’ Foscari University of Venice, Via Torino 155, 30172 Venice, Italy

**Keywords:** small microplastics, plastic additives, road dust, tires, micro-FTIR

## Abstract

Road dust is one of the environment’s most important microplastic and plastic additive sources. Traffic vehicles and the wear of tires can release these emerging contaminants, which can be resuspended in the air and washed off by stormwater runoff. In this study, a concurrent quantification and chemical characterization of additives, plasticizers, natural and non-plastic synthetic fibers (APFs), and small microplastics (SMPs, <100 µm) in samples of highway road dust (HWRD) was performed. The sampling procedure was optimized, as well as pretreatment (extraction, purification, and filtration) and analysis via micro-FTIR. The average length of the SMPs was 88 µm, while the average width was 50 µm. The highest abundance of SMPs was detected in HWRD 7 (802 ± 39 SMPs/g). Among the polymers characterized and quantified, vinyl ester and polytetrafluoroethylene were predominant. APFs’ average particle length was 80 µm and their width was 45 µm, confirming that both of these emerging pollutants are less than 100 µm in size. Their maximum concentration was in RD7, with 1044 ± 45 APFs/g. Lubricants and plasticizers are the two most abundant categories, followed by vulcanizing agents, accelerators, and pre-vulcanizing retarders derived mainly from tires. A potential relationship between APFs and SMPs in the different seasons was observed, as their concentration was lower in summer for both and higher in winter 2022. These results will be significant in investigating the load of these pollutants from highways, which is urgently necessary for more accurate inclusion in emission inventories, receptor modeling, and health protection programs by policymakers, especially in air and water pollution policies, to prevent risks to human health.

## 1. Introduction

The increasing population, urbanization, and efficient mobility of people and goods worldwide have significantly contributed to enhancing the quality of transportation in the economic growth of modern society. A significant increase in the number of highway constructions, vehicles, and distance driven on roads has occurred [1]. In 2020, the European Union (EU) passenger car fleet grew to 246.3 million cars on the road [2] with a comprehensive network length of about 136,700 km [3]. While the introduction of fossil fuel-powered motor vehicles created an economic revolution, millions of vehicles worldwide have resulted in a diffuse environmental impact, including hazards to human health. For instance, from 2014 to 2020, bad-quality air standards were linked to dense traffic near main roads and highways, since traffic is responsible for most of the exceedance of PM10 values registered in European countries [4]. In addition, there has been direct release of a wide variety of pollutants from traffic sources (and from other anthropogenic activities or volcanic eruptions, soil erosion, sand and rock, aeolian process) into the atmosphere, a mixture of combination of organic and inorganic particles defined as urban road dust (RD; [5]). The RD, which continuously accumulates on highways due to the continuous traffic of vehicles, is defined as highway road dust (HSW; [6,7]). Then, the HWRD can be suspended by vehicles and winds, dispersed into the air, or washed off by a stormwater sewer system through surface runoff. This runoff can finally end up in receiving waters (e.g., streams, rivers, lakes, and estuaries), posing a risk to aquatic and terrestrial biota [8].

The wear of tires, bitumen, road marking paints used in road pavement, brake wear, vehicle emissions, soil from construction sites, atmospheric deposition, plastic or other materials debris, and road pavement made of recycled plastics are generally deposited in the HWRD [9,10,11]. HWRD is a composite miscellany of particles, and it is considered one of the primary sources of microplastics (MPs) in the atmosphere [10,11]. Although MPs in HWRD have been detected worldwide [10,12], those MPs below 100 µm (SMPs) are almost overlooked [6]. Once in the environment, MPs can be ingested by biota [13,14,15]. SMPs can be easily ingested by invertebrates by the size of their mouthparts; therefore, they can enter the food web and be subject to biomagnification, also posing a risk to human health [16]. Additives, plasticizers, and natural and non-plastic synthetic fibers (APFs) are necessary for the production of plastic objects [17] and can be dispersed into the environment due to aging and weathering as MPs and SMPs, potentially exerting toxic effects and thus becoming a source of health risk to animals and humans [18,19,20]. The most employed APFs are stabilizers, antioxidants, UV stabilizers, plasticizers, fillers, vulcanizing agents, pigments, colorants, and reinforcements. In HWRD, tires are a major source of APFs, e.g., vulcanizing, lubricant, curing accelerators, or antioxidant additive categories [21]. The smaller the plastic particle, the bigger the active surface areas that facilitate the leaching, adsorption, and transfer of these additives throughout the entire life cycle of plastics, enhancing their negative impacts [22,23,24]. However, APFs’ pathways and the role of their transformation products under degradation or weathering are still unknown [25,26,27]. Additionally, the total budget load of SMPs and APFs in RD is still unknown, because there is a lack of standardized sampling procedures, pretreatment, and analytical methods ([16,28,29]. For complex environmental matrices such as HWRD, the pretreatment procedures (extraction and purification) are essential to avoid organic and inorganic interferents for the unambiguous identification of SMPs and APFs. The road’s remaining constituents, e.g., aggregated bitumen, oil residues, and exhaust additives, could impact the chemical identification of SMPs and APFs, underestimating their quantification [30,31]. On the other hand, the use of strong and aggressive reagents (i.e., acids or alkaline reagents) together with the use of higher temperatures can improve the denaturation/degradation of these contaminants, affecting the particles size, shape, and their chemical identification [32,33,34].

The first goal of this study was to investigate the occurrence of SMPs and APFs in the HWRD and whether these pollutants might accumulate during dry periods and be washed off by rainfall events. Another of the study’s goals was to optimize a sampling procedure for HWRD in order to collect significant samples of road dust during the 2021–2022 period. Additionally, this study focused on optimizing and harmonizing the oleoextraction procedure and purification previously developed [33] to extract SMPs and APFs from HWRD simultaneously, and the analytical method via Micro-FTIR for the simultaneous quantification and optimal identification of the pollutants under exam. Data from this study will allow for an assessment of the impact of a highway in terms of the contribution of these pollutants, a deeper understanding of the potential sources, and the design of effective and practical mitigation actions and solutions to prevent the control and reduction of these pollutants in the atmospheric, terrestrial, and aquatic environments.

## 2. Materials and Methods

### 2.1. Sampling

**Figure 1:**
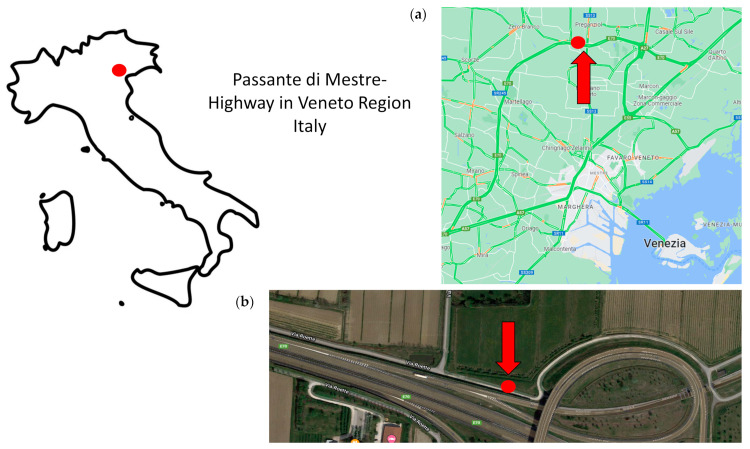
HWRD samples were collected from a highway 32.3 km long, namely, Passante di Mestre, on the mainland near Venice in Italy (Casale sul Sile, Treviso, Italy), as highlighted in the map (**a**). In picture (**b**), the red dots highlighted the sampling site.

HWRD samples were collected during dry periods (at least 2 weeks after a rainfall event) from winter 2021 to winter 2022 from a highway 32.3 km long, namely, Passante di Mestre, on the mainland near Venice in Italy (Casale sul Sile, Treviso, Italy; Figure 1). From summer 2021 to winter 2022, HWRD samples were also collected to evaluate a possible spatial variability along the same highway. Before sampling, several investigating explorations were performed to choose the position of sampling from the edge of the highway, considering slope, distances from the drains, technical operations, and safety access during traffic activities. Also, meteorological factors were taken into account before the sampling (e.g., windless day and no rain/mist conditions), and the sampling operations were carried out far from road cleaning operations [35].

Three areas of one square meter each of the highway surface were selected to collect average composite HWRD samples representative of each event and signed with steel tape. The three areas were three meters away from each other and one meter from the highway edge. Inside each square meter, the HWRD was collected using a brush made of natural coconut fibers and a previously decontaminated steel dustpan; the surface was brushed for about 15 min for each area to collect approximately a minimum of 30 g per area (Figure 1). The average composite HWRD sample was then transferred to decontaminated glass jars covered with aluminum foil, weighed, and transported to the laboratory. Technical operations, authorization access, and remote control were supported by StormWater Italia (SWI—Marghera-Venice) company and CAV (Concessioni Autostradali Venete) of Venice, Italy.

### 2.2. Reagents

Ultrapure water (UW) was produced by a UW system (Elga Lab Water, Veolia, High Wycombe, UK).

The reagents employed were hydrogen peroxide (H_2_O_2_, 30%, ACS reagent, Sigma Aldrich, Merck, Darmstadt, Germany), hexane (puriss. p.a., ACS reagent, reag. Ph. Eur., ≥99% (GC) Sigma Aldrich, Merck, Darmstadt, Germany), ethanol (absolute, for HPLC, ≥99.8%, Sigma Aldrich, Merck, Darmstadt Germany), and methanol (for HPLC ≥ 99.9% Sigma Aldrich, Merck, Darmstadt, Germany). The oil employed in the oleoextraction was an organic solvent-free and cold-pressed sunflower seed oil (SSO, Crudolio, Camisano Vicentino (VI), Italy, density 0.918 g/cm^3^).

Aluminum oxide filters (0.2 μm, 47 mm diameter, ANODISC (Anopore Inorganic Membrane Whatman) were purchased from Merck (Merck, Darmstadt, Germany).

Silver-gray particles of polyamide 12 (PA 12, range 40–250 µm, average size 90 µm) were purchased at Goodfellow GmbH (Hamburg, Germany).

### 2.3. QA/QC

A detailed protocol was designed to minimize potential plastic contamination of samples during sampling, transport of samples, pretreatment, and analysis. Sampling activities were performed upwind. During sampling, the operators wore cotton lab coats, nitrile gloves, and cotton boot covers to protect against contamination from plastic fibers, rubbers from shoes, and other contaminants from the operator. The HWRD samples were collected carefully in previously decontaminated glass jars covered with aluminum foils until they arrived at the plastic-free Clean Room ISO 7 [36]. This laboratory is a controlled environment entirely made of stainless steel, where there are no plastic materials, even in the air prefilters. To minimize contamination, pretreatment procedures (i.e., extraction, purification, and filtration) were carried out under a decontaminated steel fume hood in the plastic-free Clean Room ISO 7. Also, during all the pretreatment procedures, the operators wore cotton lab coats and nitrile gloves. Only decontaminated glassware and steel tools are employed in the cleanroom to minimize/avoid contamination. All the steel and glassware (including the glass jars for sampling) were previously rinsed with UW, decontaminated with methanol, a 50% (*v*/*v*) methanol and ethanol solution, and finally with ethanol. After filtration, all filters were stored in decontaminated glass Petri dishes covered with aluminum foil. Before the analysis, filters were transferred from the fume hood in the cleanroom to the micro-FTIR laboratory and carefully covered with aluminum foil to avoid any external contamination. Reagent blanks (i.e., ultrapure water, methanol, ethanol, H_2_O_2_) and procedural blanks were tested for SMPs or APFs. A recovery test was performed by spiking replicates of one of the HWRD samples under exam with silver-gray PA 12 particles.

### 2.4. Oleoextraction, Purification, and Filtration of RD Samples

In this work, the pretreatment procedure, i.e., the oleoextraction and purification procedure employed for HWRD samples, was optimized from the procedure previously developed for sediments by Corami and coworkers at the Institute of Polar Sciences CNR-ISP [33].

Several tests were performed. For this purpose, the average composite HWRD sample from the first campaign was homogenized by quartering and subsampled into aliquots. Tests were run in triplicate. In the first test, the HWRD aliquots (5 g each) were placed inside a decontaminated Erlenmeyer flask. Then, 100 mL of UW and 5 mL of H_2_O_2_ was added to pseudo-digest the organic matter and other organic interference from the road in the samples. The Erlenmeyer flasks were stirred at room temperature for 3 h on a multipurpose orbital shaker. Then, the slurry was poured into a 500 mL separating funnel, adding 7 mL of sunflower seed oil (SSO, Crudolio, Camisano Vicentino (VI), Italy) and filled with 300 mL of UW. The separating funnels were stirred for 15 min at 100 rpm on an orbital shaker to form an emulsion and left to rest for 24 h for the complete separation of the three phases (i.e., HWRD, aqueous, and oil phases) to extract SMPs and APFs. After that time, all the oil phases (where the SMPs and APFs were) and almost 300 mL of the aqueous phase were carefully poured into a second previously decontaminated separating funnel, taking care not to dislodge the settled HWRD. Then, 10 mL of H_2_O_2_ was added to the second separating funnel, which was stirred again and left to rest for another 24 h for the complete separation of the two phases. Then, the water was discarded, and the oil phase was recovered with 20 mL of hexane and 20 mL of ethanol and poured into a previously decontaminated Erlenmeyer flask.

In the second test, three aliquots (10 g each) of the same HWRD sample were collected and placed inside three decontaminated Erlenmeyer flasks, to which 10 mL of H_2_O_2_ was added with 100 mL of UW. The oleoextraction was performed as in the first test. The third test differed from the second only in the first rest time, which increased from 24 to 72 h. The oleoextraction procedure of the third test was adopted for all samples of HWRD since it was the optimal test.

The oleo-extracts were filtered under the fume hood using a decontaminated glass vacuum filtration system (VWR International, Milan, Italy) and aluminum oxide filters. Purification was performed during the filtration. Briefly, filters were rinsed following the procedure developed by Corami et al. [33,37,38], alternating a 70% solution of ethanol–methanol, ethanol, and UW and ending with ethanol (ratio 2:3:1). The oleo-extracts were then filtered alternating hexane, 70% solution of ethanol–methanol, and ethanol. After having filtered all the oleo-extract, the filter was rinsed alternating UW, a 70% solution of ethanol and methanol. Reagent and procedural blanks were pretreated and filtered in the same way. After filtration, filters were left to dry in the clean room for at least 72 h at room temperature in previously decontaminated glass Petri dishes, which were covered with decontaminated aluminum foil. The filters were then transported inside decontaminated Petri dishes to the micro-FTIR laboratory and analyzed.

### 2.5. Quantification and Chemical Identification of SMPs and APFs Using Micro-FTIR

Quantification and simultaneous particle identification were performed using a micro-FTIR Nicolet™ iN™ 10 (Thermo Fisher Scientific, Madison, WI, USA), using an ultrafast motorized stage and liquid nitrogen-cooled MCT detector (mercury cadmium telluride detector). The analysis was performed in transmittance mode employing the PARTICLES WIZARD section of the Omnic™ Picta™ software (version for Windows 10) as previously described by Corami et al. [19,33,37,38]. For the quantification of SMPs and APFs, microscopic counting was performed. Since counting the entire surface area of the filter is time-consuming, the microscopic counting procedure is applied, which allows for robust, reproducible, and statistically representative quantification [33].

Briefly, at least 20 areas of known size (i.e, count fields 2000 µm × 1200 µm) were randomly chosen with no overlapping. In this work, on each count field, an average of 300 particles was selected by employing the WIZARD section; then, for each particle, 64 co-scans were collected (spatial resolution 100 μm, aperture 100 μm × 100 μm, spectral range 4000–1200 cm^−1^). For each particle selected, a spectrum was retrieved and compared to spectra in several reference libraries (the complete list of reference libraries is in the Supplementary Information, Figure S1). Further, retrieving the length and width of each particle analyzed was performed using the imaging section of the software. The length ranged from the minimum LOD (limit of detection) size of the imaging of the micro-FTIR (5 μm; [19,33,37]). Only spectra with a match percentage ≥65% (optimal identification) were considered as identified and then counted for quantification. After the identification was performed with the PARTICLES WIZARD section, the collected spectral maps were analyzed (point-and-shoot analysis) to double-check the identification. The abundance (SMPs/g and APFs/g) and the weight (μg SMPs/g and μg APFs/g)) of SMPs and APFs were then calculated according to equations modified from Corami et al., [19,33].

The abundance (SMPs/g and APFs/g) was calculated as follows:

(1) $$\frac{N}{g}=\frac{\left(n\cdot F\right)}{g}\;$$

where N = SMPs or APFs, n = number of SMPs or APFs counted on 20 fields, g = weight of the aliquot of each HWRD sample, and F = count factor, calculated as follows:

(2) $$F=\frac{filter\;area}{\left(count\;field\;area\cdot n\;count\;fields\right)}$$

where the filter area is 1734.07 mm^2^.

Aspect ratio (AR; [33,38]) is the ratio between the maximum length (L) and the maximum width (W) of the smallest rectangle (bounding box) enclosing the shape, calculated according to Equation (3):

(3) $$\mathrm{AR}=\frac{L{\;}_{}}{W_{}}$$

L and W were retrieved using the imaging function of Omnic™ Picta™ software. When AR = 1, particles are considered spherical, while when AR = 2, particles are elongated/ellipsoidal and considered elliptical. When AR ≥ 3, particles are considered cylindrical. Volumes of SMPs and APFs are then calculated according to their geometrical shape (i.e., sphere, ellipse, and cylinder).

The weight (µg/g) of each polymer or plastic additive is calculated according to the Equation (4):

(4) $$w=\frac{V\cdot \rho \cdot F}{g}$$

where V= volume of each particle, ρ= density of each particle, and F = count factor

The total weight of SMPs (µg SMPs/g) and APFs (µg APFs/g) is the sum of all particle weights.

The number of dry days was retrieved from the Regional Agency for Environmental Protection in Veneto (ARPAV 2022) networks from the Favaro Veneto station [39]. (https://www.arpa.veneto.it/bollettini accessed on 2 June 2022).

### 2.6. Statistical Analyses

SMPs’ and APFs’ abundance data follow a Poisson distribution, and Poisson’s confidence interval was calculated accordingly [19,33,38]. Statistical analyses were performed using STATISTICA software (ver. 14.0; TIBCO, Palo Alto, CA, USA). The homogeneity of the variances of SMP and APFs abundance was tested (Fisher’s exact test, F test; α = 0.05). After ascertaining the non-homogeneity of the variances, non-parametric statistical tests were performed. The Kruskal–Wallis test (*p* < 0.05) was employed for multiple comparisons.

## 3. Results and Discussion

### 3.1. Optimization of the Pretreatment (Oleoextraction, Filtration, and Purification)

Studies on RD and HWRD are scarce. Different sampling procedures, pre-treatments, and analytical techniques have been employed. Regarding the sampling procedure, tools, e.g., pans, brushes, vacuum cleaners, and brooms, were investigated, while meteorological parameters and land use for different kinds of RD were considered [35]; however, the entire detailed procedure and QA/QC protocol (especially the blanks) is often lacking or missing. In this study, we used brushes of recognizable natural fibers and steel dustpans, decontaminated prior to use. Additionally, we paid much attention to the mode of collection, both in the positioning of the operator and in the clothing, and in the collection of blanks (i.e., field blanks, reagent and procedural blanks) to minimize possible contamination. For the pretreatment procedures, density separation was the most common method employed (e.g., saturated sodium chloride solution [40], or zinc chloride solution [41]; however, this procedure allows the collection of polymers only in a specific density range. Staining techniques were used to analyze MPs in RD [42,43,44]; however, MPs were not unmistakably discriminated from the other particles in the RD. Additionally, salts used for the flotation are interferents for the analysis via spectroscopic techniques, which are necessary for unambiguously identifying the particles.

**Figure 2:**
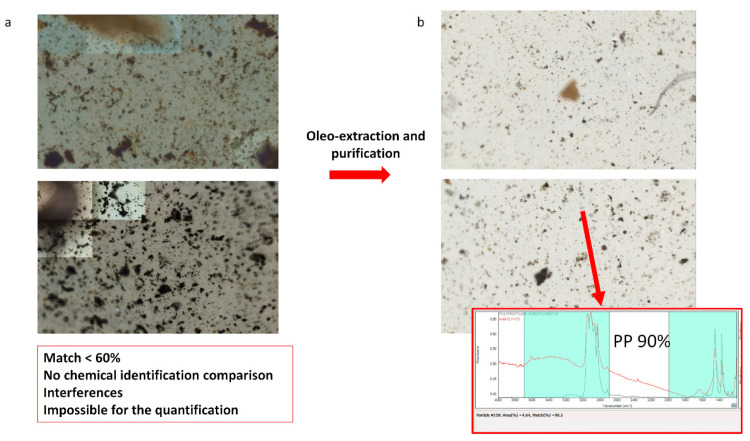
Count fields obtained via micro-FTIR before (**a**) and after (**b**) the optimization of the oleo-extraction resulted in a change from suboptimal identification (some interferents, e.g., different aggregates of oil, bitumen, and other organic interferents still coated the particles under examination) to optimal identification for SMPs and APFs.

Oleoextraction is a method employed for different environmental matrices [33,45,46,47,48], such as soils and sediments, and it is straightforward and efficient concerning time, cost, health, and environmental risk since no hazardous salt solutions are needed [49]. The oleoextraction procedure we developed [33] for the simultaneous extraction of SMPs, APFs, and all the other microlitter components of a wide range of densities was optimized for HWRD. The purification procedure previously developed [33,37] was successfully employed for other environmental matrices, proving essential to clean the SMPs and APFs from all the interferents and allowing optimal identification [19,33,37,46]. Indeed, the interferents’ presence limits the analysis’s applicability, and MPs and APFs < 100 µm are usually neglected [42]. Optimization of the oleoextraction resulted in a change from suboptimal identification, as some interferents (e.g., different aggregates of oil, bitumen, and other organic interferences) still coated the particles under examination (Figure 2a from the first test) to optimal identification for SMPs and APFs (Figure 2b from the third test), with an average optimal identification match of 87% for SMPs and 89% for APFs (see supplementary information for best spectra in Figure S1) in the third test. Therefore, due to better spectral identification, the SMPs’ and APFs’ abundance and distribution improved from the first to the third text, avoiding potential underestimation. Hence, the oleoextraction procedure in the third test was optimal and applied to all the other samples. No SMPs or APFs were observed in reagent or procedural blanks. The oleoextraction procedure used in the third test was employed for the recovery tests. The method’s yield was excellent, exceeding 90% (92%). Thus, this pretreatment method was accurate, efficient, and reproducible.

### 3.2. Quantification and Simultaneous Identification of SMPs in HWRD Samples

**Figure 3:**
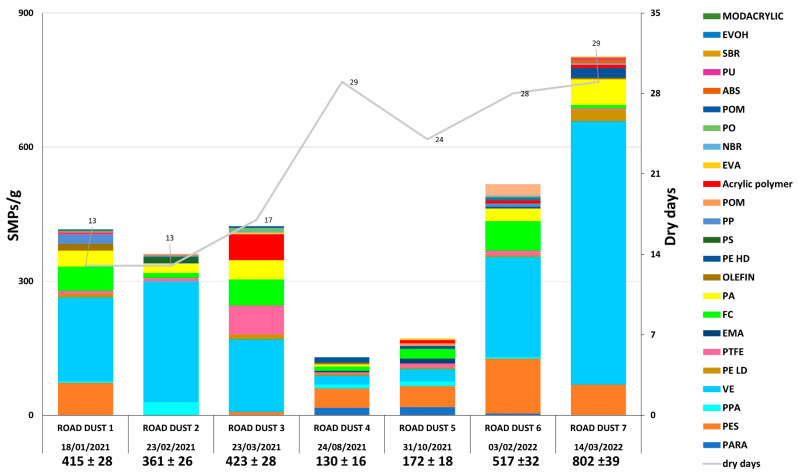
Abundance and polymer distribution of SMPs in HWRD samples analyzed (SMPs/g). The number of dry days before each sampling campaign is reported for each sample collected.

The complete list of the identified polymers and their relative acronyms is reported in Table 1. The abundance of SMPs is reported with the respective confidence limit (error) and respective dry days since rainfall events (Figure 3). The trend shows a decrease in the summer of 2021 (i.e., the minimum value was in HWRD 4 (130 ± 16 SMPs/g)) compared with samples taken in the two winters. The concentration increased in the fall and late winter, where its highest value was found (HWRD 7, 802 ± 39 SMPs/g). The same trend was observed for the weight (Figure S2; the maximum value in HWRD 7 (393.7 ± 27.50 µg SMPs/g) and the minimum value in HWRD 4 (16.61 ± 5.65 µg SMPs/g, respectively). The differences observed among the samples were statistically significant (Kruskal–Wallis test, *p* < 0.05 for both abundance and weight). Differences between winter and summer in HWRD can originate from road cleaning maintenance, different input from traffic vehicles, highway characteristics, and duration and intensity of precipitation [6,50]. Also, wet and dry depositions might play a role in SMP abundance on the highways and roads during different seasons due to distinct anthropogenic sources, such as domestic or industrial activities. However, these seasonal differences are difficult to explain and compare with scientific literature because of limited data for the particles under exam in this matrix. 

This study was focused on achieving an accurate and efficient method for quantifying and concurrently identifying SMPs in this environmental matrix for the first time. Data on the atmospheric compartment, seasonal variations, and correlation with meteorological, traffic, and other parameters in the HWRD are not that common in research; these shortcomings represent the main perplexities in the modeling analysis of stormwater and HWRD [11,51]. Therefore, these first data are the basis for gathering information to provide accurate and appropriate models, ultimately achieving reductions in the rate of pollution from highways and roads through the implementation of a combination of regulatory policies and management actions [52]. Future studies will aim to increase the number of samples collected throughout the seasons to fully understand which variables affect the distribution of SMPs.

**Figure 4:**
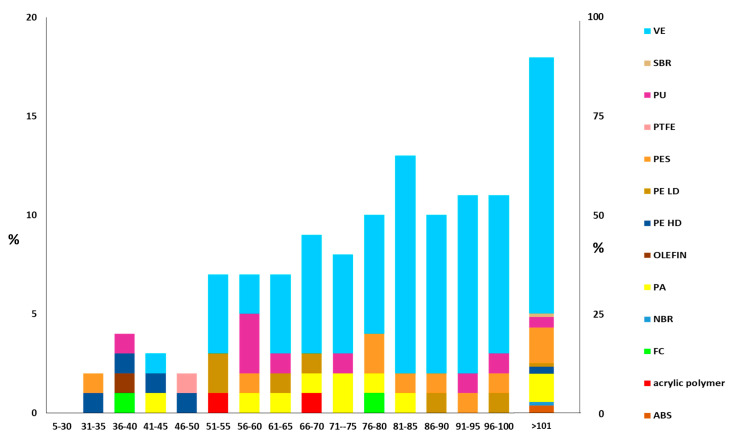
SMP size distribution in HWRD samples analyzed.

Regarding the SMP size, the average length of the particles was 88 µm, while the average width was 50 µm, with ellipsoid (extrapolated from AR, Figure S3) being the most common shape for SMPs in HWRD. Regarding the SMP size distribution (Figure 4), a large portion of SMPs were ≤50 µm. A majority of polymers were middling in size (e.g., VE), while others were very small (e.g., olefin, acrylic, or PTFE) or very large (e.g., ABS or NBR). Due to the small sizes observed, these particles may have originated on the surface of the highway and could be easily transported by suspension in the air or washed off by highway stormwater runoff (HWS), and they may not be effectively trapped in the environment by stormwater treatment plants. In a previous work [46], HSW runoff was collected and analyzed for the abundance and distribution of SMPs and APFs collected in the same site during winter 2021. It should be highlighted that the majority of SMPs in HSW was <100 µm and the length ranged from 25.0 μm to 80.4 μm. Compared with HSW, in HWRD samples, there is a slightly higher presence of plastic particles < 100 µm; one hypothesis could be that contributions from vehicles and other highway sources may give rise to these particles, which were then more challenging to transport through stormwater runoff and could be persistent on the highway surface because of their weight and size. However, a high number of SMPs ≪100 µm were found in both matrices; once dispersed in the environment, these particles can quickly enter the trophic web by being ingested by invertebrates, posing a threat to them and other organisms at higher levels. The distribution provided in this study could improve knowledge of emerging pollutants associated with this complex matrix to study their emission factors and physicochemical profiles, which are urgently needed for more accurate inclusion in emission inventories, receptor modeling, and health protection programs by policymakers, especially in air and water pollution policies, to protect human health [53].

VE and PTFE are two of the most abundant polymers in HWRD samples, as per the HSW runoff composition analyzed in the previous work. Hence, these polymers could originate from vehicular traffic, from tires to car chassis, seats, cooling systems, and vehicle parts (Rosso et al., 2022). The oleoextraction procedure allowed the optimal identification of high-density polymers, such as PTFE and FC. VE was identified in a larger size than the other typologies; hence, it could be less subjected to further degradation once emitted in the asphalt, maybe due to its high density. However, more investigations are needed to better understand the correlation between SMP size and typology in HSWR. PA 6 (commonly named nylon) was identified in all samples in high amounts. This polymer is often overlooked in environmental studies because strong oxidants or temperatures higher than the polymer’s glass transition temperature (55–60 °C; [33,37,38] are employed (80 °C [43]; 75 °C [44]; from 85 °C to 110 °C [10]). PA6 was observed in the different size groups, from the smallest to the largest (>100 µm), validating their correct and optimal identification in each size distribution range.

Acrylic and PES, with a minor part of PU, can be used for road-marking paints (RMPs). In European countries, it is estimated that more than 50% of road dust-associated MPs originate from RMPs, which, together with tire wear particles, are released into the water environment every year [6]. Most of the RMPs detected in the environment were the smallest detected, and they mainly result from the degradation by UV irradiation and flushing by rainwater [54]. Compared to the other polymers, PES and acrylic identified in HSRD samples were small (most less than 40 and 70 μm, respectively), suggesting that their potential common input source can be RMPs. RMPs’ hazard is mainly connected to their toxin-resistant bacteria composition, which can be transported into the environment and ingested by biota. Since data on them are still limited, quantifying RMPs in environmental samples is crucial [54].

EVA and ABS identified in HWRD samples are used as asphalt modifiers, and they could potentially be released from plastic-modified road materials into road dust [6,42]. HDPE, LDPE, PP, and PO were also identified in HWRD samples. These polymers can be released from decomposed recycled plastics (plastic bottles and bags, bottle caps, nets, and food containers) often recovered in the curbside [6]. It was notable that on the highway edge where the sampling was carried out, a high amount of plastic litter was always found, despite the efficient cleaning maintenance of the highway.

The comparison of our results with other studies on RD is quite challenging due to the different units of measure, pretreatment methods, and analytical techniques employed. While those studies analyzed MPs in RD from urban or rural areas, those in HWRD are mostly overlooked. In one of the first studies on RD, the MPs’ abundance averaged around 8760 MPs g^−1^ [55] and the particles were well above 100 µm. In another study, Nile red dying was employed to analyze MPs in RD from a highly industrialized area, and the average amount was 265 ± 78 MPs [44]. Using separation from flotation, MPs’ abundance in Australia ranged from 2.060 to 52.93 MPs g^−1^, while the particle size ranged from 80 to 4700 µm, with an average size of 1200 µm [40]. From roads in Goyang City, South Korea, MPs in RD ranged from 552 (±39) g^−1^ to 1530 (±602) MPs g^−1^ in different dry periods, while the particle size ranged from 100 µm to far above 2000 µm [41]. MPs’ abundance was 52 ± 13 MPs g^−1^ and 113 ± 25 MPs g^−1^ in residential and industrial areas, respectively, and most of the particles were <2000 µm [6]. In most of these studies, polymer identification was not provided, the QA/QC detail protocol was not sufficiently described, or stronger pretreatments were employed, leading to unsuccessful identification of polymers (e.g., some studies explained that they obtained poor-quality spectra, being lost or damaged). Furthermore, in other studies, the unit of measurement was different (MPs m^−2^), and the particles were all above 200 µm, so SMPs were neglected [56]. Speaking of seasonal differences may be somewhat difficult, as pointed out by a study in urban RD in China [57], where the dominant MPs were in the range 500–1000 µm, and only a tiny portion was in the range 150–250 µm, neglecting those below 100 µm. Additionally, the frequent cleaning of the urban roads prevented the accumulation of MPs in RD during the antecedent dry days. However, MPs in HWRD remain poorly investigated. Due to the lack of standardized pretreatment and analytical methods, there is a need to investigate the abundance and distribution of SMPs in HWRD and urban RD, considering factors such as the anthropogenic inputs, high traffic density, frequency, etc., but also the fragmentation of plastic litter on roads and the wind intensity and direction, which are factors not explored to date [41,57].

### 3.3. Quantification and Identification of APFs in HWRD Samples

**Figure 5:**
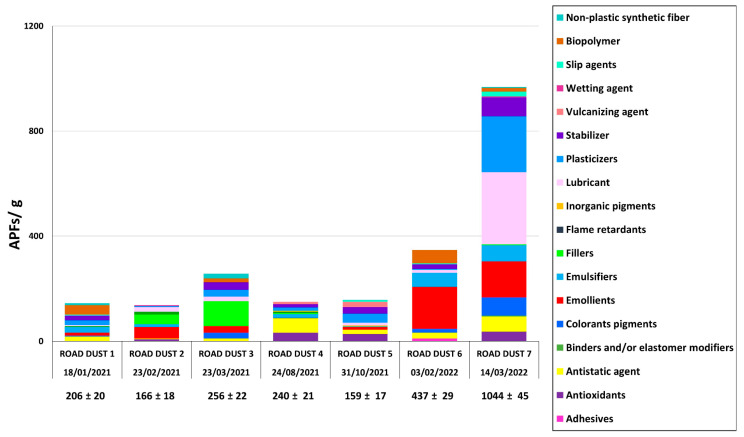
Abundance and distribution of APFs in the samples of road dust analyzed (APFs/g). APFs are grouped according to their function.

Simultaneously with the quantification and identification of SMPs, APFs were identified and quantified in all HWRD samples. The complete list of identified APFs and their acronyms is reported in Table 1. Due to the high amount of APFs identified, they were catalogued into classification groups of curing agents, antioxidants, fillers, vulcanizers, etc. (Figure 5). Since some APFs could be used for various purposes, including additives for plastic polymers, the classification was scrupulously employed by comparing those APFs identified with the limited existing literature [58,59]. The entire list of all singular APFs with their abundance is also shown in the supplementary information (Figure S4). As already observed for SMPs, the APF trend showed an increment in the late winter of 2022, when HWRD7 showed the maximum abundance and weight of APFs (1044 ± 45 APFs g^−1^ and 249.2 ± 21.88 µg APFs g^−1^, respectively). The minimum abundance was observed in HWRD5 (159 ± 17 APFs g^−1^) during the summer of 2021, while the minimum weight was HWRD2 in the winter of 2021 (4.891 ± 3.061 µg APFs g^−1^; Figure S5 in the Supplementary Information).

According to the Kruskal–Wallis test (*p* < 0.05), the differences in APFs’ abundance observed during the three sampling seasons are statistically significant. A potential relationship between the trends of APFs and SMPs in the different seasons may be related; however, other further statistical and modeling analyses should focus on the relationship with other meteorological and environmental factors affecting HWRD sources and pathways.

Regarding size, most of the APFs in the HWRD samples were far below 100 µm and similar to the sizes observed in HWSW ([46]; as a matter of fact, most of the particles were in the range of 36–90 µm, with an average length of 80 µm and average width of 45 µm, slightly lower than those SMPs. As already observed for SMPs, ellipsoid (calculated by AR) was the most common shape (Figure S6). Due to their size, once spread in the environment, APFs can be resuspended by winds and atmospheric currents or can enter the trophic network through ingestion by invertebrates.

Very few studies investigated the presence of APFs in HWRD; hence, comparison with other studies is critical. As already observed for SMPs, different analytical techniques are employed for quantifying these additives, and micro-FTIR spectroscopy is still limited. For instance, a study analyzed different APFs with GC–MS and MPS (>150 μm) in RD from Japan [12]. DA was also observed in our samples and catalogued as a plasticizer. It is a cold-resistant plasticizer for PVC resin. PVC in the highways could originate from pipes or delineators, which may not be fragmented in particles below 100 µm. However, these plasticizers can be a good proxy for the PVC source in HWRD. Besides the different analytical techniques employed, the impacts of these APFs are mostly unknown. Some studies highlight their potential toxicological effects on biota due to leaching, and therefore studying their sources and pathways in the environment is necessary. For instance, APFs leaching from tires negatively impact marine phytoplankton growth, the base of marine food webs [26]. Data on APFs are essential to evaluate emission factors and understand the physicochemical profiles of RD, preventing biota impacts and human health [53].

There are various categories of APFs, which could originate from plastic litter accumulated in the highway curbside or from vehicles such as cars, buses, and lorries, suggested emission of these pollutants by leakage of hydraulic fluids and motor and transmission oils [60]. From these first results, lubricants and plasticizers are the two most abundant categories of APFs. These additives are most commonly used for packaging materials and also to give specific properties to plastic objects [58]. For instance, elastomers need plasticizers to become softened [61].

Another relevant source of APFs in HWRD could be their emission from tire wear particles on the highway asphalt. This takes into account the presence of vulcanizers, accelerators, and pre-vulcanizing retardants for rubbers and elastomers in HWRD. For instance, 5-MBTR is an antiozonant to prevent rubber degradation, according to our previous study on HSW [47]. Antiozonant E-9604^®^ and Naugatuck Antioxidant 451^®^ were also identified and quantified. These antioxidants prevent or retard damage caused by ozone, and especially prevent degradation of elastomers for SBR and NBR [62] Hence, they can be derived from the automotive industry and tires. Also, dicumyl peroxide (DCP) is a cross-linking agent employed for tire rubber [63,64]. and for EPDM and NR blends to improve tensile properties, heat resistance, gel content, and morphology [65].

Non-plastic synthetic and natural fibers, e.g., rayon, were also identified and quantified. Besides cigarette butts, rayon could be used as an additive in tires to maintain structural integrity and resist mechanical wear and tear [47].

## 4. Conclusions

To the best of our knowledge, the novelty of this study is the concurrent identification and quantification of SMPs and APFs in HWRD. APFs can be suitable tracers for determining the potential sources of SMPs in the environment and to gain a comprehensive overview of the whole highway system. The accurate and efficient pretreatment procedure was optimized to achieve a simultaneous chemical characterization and quantification of these emerging pollutants via micro-FTIR spectroscopy. Vehicular traffic, packaging materials, and the wear of tires are supposed to be the major sources of SMPs and APFs in HWRD. Future studies need to focus on the correlation between meteorological and traffic parameters. SMPs’ size distribution confirmed that most of these particles were less than 100 µm; this fact highlights their potential transport through the atmospheric compartment by the resuspension or being washed off by stormwater runoff. The results will be significant to improve knowledge on these pollutants in HWRD, which can then be run off by stormwater and dispersed into the environment. This is necessary for potential strategic solutions by the authorities and decision-makers to monitor and manage the quality of urban air and water policies to prevent damage to biota and human health.

## Figures and Tables

**Table 1:** The complete list of identified SMPs and APFs and their acronyms.

**SMPs**	**Abbreviations**
Acrylic polymer	Acrylic polymer
Acrylonitrile butadiene styrene	ABS
Ethylene methyl acrylate	EMA
Ethylene-vinyl acetate	EVA
Ethylene vinyl alcohol	EVOH
MODACRYLIC	MODACRYLIC
Nylon	PA
Olefin	OLEFIN
Polyarylamide	PARA
Polyethylene HD	PE HD
Polyethylene low density	PE LD
Polyester	PES
Polyolefin	PO
Polyoxymethylene	POM
Poly(p-phenylene oxide)	PPE
Polyphthalamide	PPA
Polypropylene	PP
Polystyrene	PS
Polytetrafluoroethylene	PTFE
Polyurethane	PU
Styrene butadiene rubber	SBR
Vinyl ester	VE
**APFs**	**Abbreviations**
(N-(2-ethoxy phenyl)-N-(2-ethyl phenyl)-ethanediamide)	2E2ANI
1,7,7-Trimethylbicyclo[2.2.1]heptan-2-one	1,7,7-TMBC-2,2,1-H
2-(2-phenylpropan-2-ylperoxy)propan-2-ylbenzene (dicumyl peroxide)	DCP
2-methoxyethyl stearate	MS
50% active glycerol monostearate	AGM
5-methyl-1h-benzotriazole	5-MBTR
Adipic 1,3-butylene glycol polymer	ABGP
Antiozonant e-9604^®^	Antiozonant E-9604^®^
Bis(2-hydroxyethyl dimerate	BHD
Butyl (Z)-octadec-9-enoate	BO
Butyl benzyl sebacate	BB
Butyl epoxy stearate	BE
Butyl ricinoleate	BR
Butyl vinyl ether	NBVE
Calcium pelargonate	CP
Calcium stearate	CS
Calcium sulfonate	CS
Calcium zinc molybdate	CZM
Chloroalkyl phosphate ester	CPE
Cocoamidopropyl betaine	CAPB
Cyanox STDP antioxidant	CYANOX STDP Antioxidant
Dibasic lead phosphite	DLP
Dicapryl maleate	DOM
Dicetyl maleate	DM
Diester of 3-dodecylthio propionic acid and tetraethylene glycol, hydrated amorphous silica	3-DTPA-DE/TEG
Dihydrogen phosphate;2-hydroxyethyl-dimethyl-[3-(octadecanoylamino)propyl]azanium	2-HE2M-3-OAPAP
Dimethyl sebacate	DMS
Dimodan lsk	
Dioctyl adipate	DA
Dipropylene glycol dinonanoate	DGD
Ethylene glycol monooleate	EGM
Fatty acid ester	FAE
Glutaric acid-based monomeric ester	GAM
Glyceryl monoricinoleate	GMR/SSL
Glyceryl trioleate	GT
Ground calcium carbonate #1	GCC
Isooctyl stearate	IS
Methoxyethyl acetyl ricinoleate	MAR
Methyl octadecadienoate (methyl (9z,12z)-octadeca-9,12-dienoate-metyl linoleate)	Mlo
Methyl oleate	MO
Methyl ricinoleate	MLO
Methyl tallowate	MT
Microcrystalline wax	MCW
Montan ester wax mixture	MEW
Naugatuck antioxidant 451®	Naugatuck Antioxidant 451®
Neopentyl glycol	NG
Octadecanoic acid, calcium salt	OC SALT
Octyl dipropionate	OD
Organo quaterarny antistatic agent	QAC
Pentaerythritol monoricinoleate	PM
Peptizer 566	Peptizer 566
Phthalocyanine	Pc
Plasthall p-670 (polyester adipate)	PEA
Polymeric epoxy plasticizer	PEP
Propylen glycol dilaurate	PGDL
Quaternary ammonium compound	QAC
Rayon	RAYON
Resinall cp-25	Resinall CP-25
sebacic acid polymer with a polyol and vegetable oil	SAP
Sodium salt of alkyls sulfonic acid in polyolefin	SSAS in PO
Sorbitan isostearate esters	SIE
Sorbitan laurate	SL
Sorbitan monostearate	SL
Sorbitan trioleate	STO
Tetrahydrofurfuryl oleate	TO
Triglycerol diisostearate	TD
Unsaturated distilled monoglyceride	UDM
Vanox sn-1	Vanox SN-1
WB7 (titanium dioxide)	TiO_2_

## Data Availability

Data are contained within the article.

## Supplementary Materials

The following supporting information can be downloaded at https://www.mdpi.com/article/10.3390/toxics11110936/s1. Figure S1: Some of the best spectra of the polymers observed in the samples. A match percentage higher than 65% indicates that the polymer spectrum was optimally identified; Figure S2: Weight of SMPs(µg/g) in the HWRD samples analyze; Figure S3: Ellipsoid (extrapolated from AR) being the most common shape for SMPs in HWRD. When AR = 1, particles are considered spherical (S), while when AR = 2, particles are elongated/ellipsoidal (E), and they are considered elliptical. When AR ≥ 3, particles are considered cylindrical (C); Figure S4: APFs’ abundance (APFS/g) with the list of all singular APFs analyzed in all the HWRD samples; Figure S5: Weight of APFs (µg/g) in all HWRD samples analyze; Figure S6: Ellipsoid (extrapolated from AR) being the most common shape for APFs in HWRD. When AR = 1, particles are considered spherical (S), while when AR = 2, particles are elongated/ellipsoidal (E), and they are considered elliptical. When AR ≥ 3 particles are considered cylindrical (C).
