# Supplementary material for: Quantification and Chemical Characterization of Plastic Additives and Small Microplastics (<100 μm) in Highway Road Dust
Source: Toxics. 2023 Nov 17;11(11):936. doi: 10.3390/toxics11110936 (PMC10674966; doi:10.3390/toxics11110936)
Supplement: Supplementary file 1 [file toxics-11-00936-s001.zip › toxics-2684482-supplementary.pdf]

Quantification and chemical characterization of plastic additives and small microplastics (<100 µm) in highway road dust

Beatrice Rosso, Barbara Bravo, Elena Gregoris, Carlo Barbante, Andrea Gambaro, Fabiana Corami

S1 List of libraries employed with Micro-FTIR

- Synthetic fibers by Microscope
- Plastic Fibers
- Polymer additives and plasticizers
- HR Polymer additive and plasticizers
- HR Hummel Polymer and plasticizers
- Common materials
- HR Nicolet Sampler Library
- HR Sprouse Polymer additives
- HR rubber compounding materials

Figures in Supplementary Materials

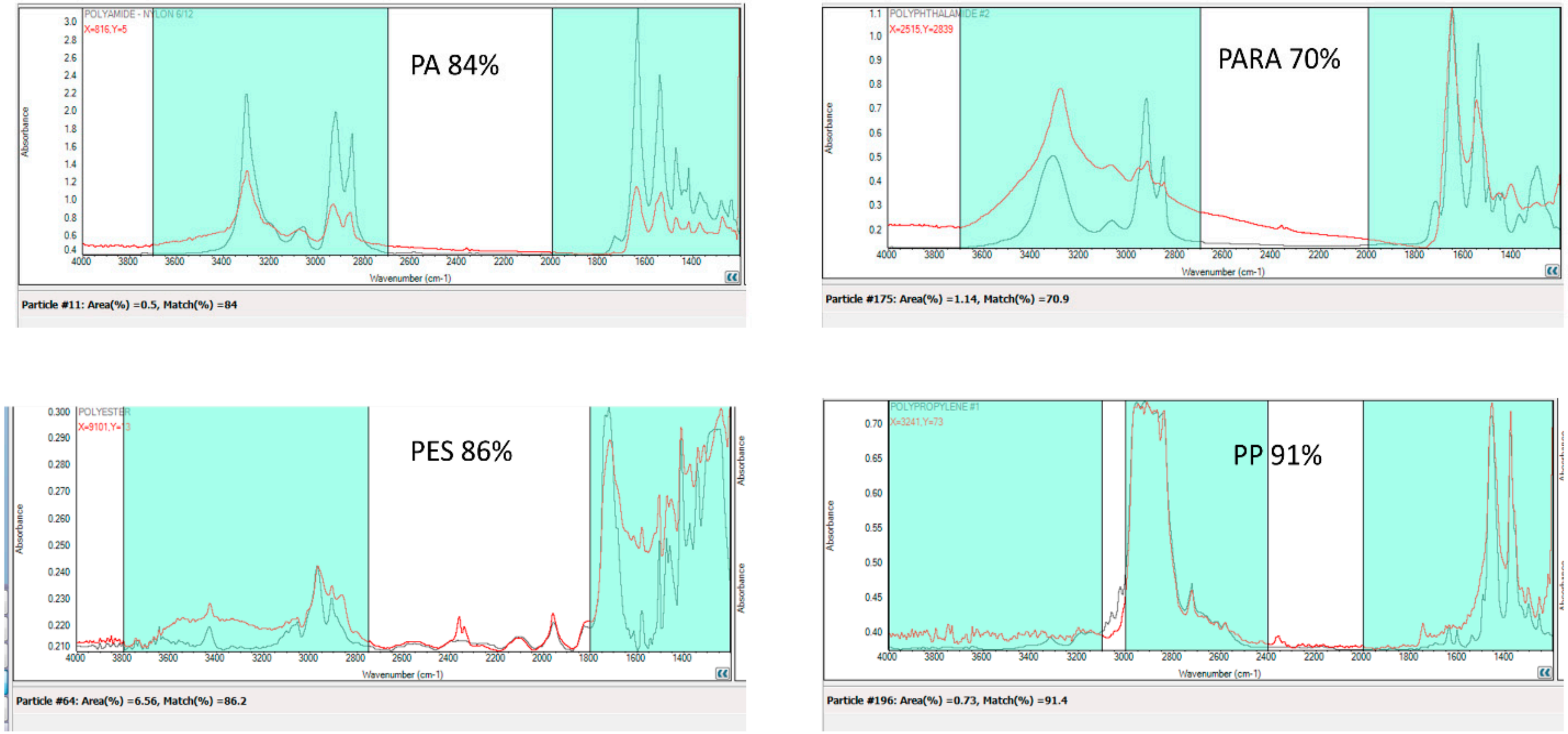

Fig. S1: Some of the best spectra of the polymers observed in the samples. A match percentage higher than 65% indicates that the polymer spectrum was optimally identified.

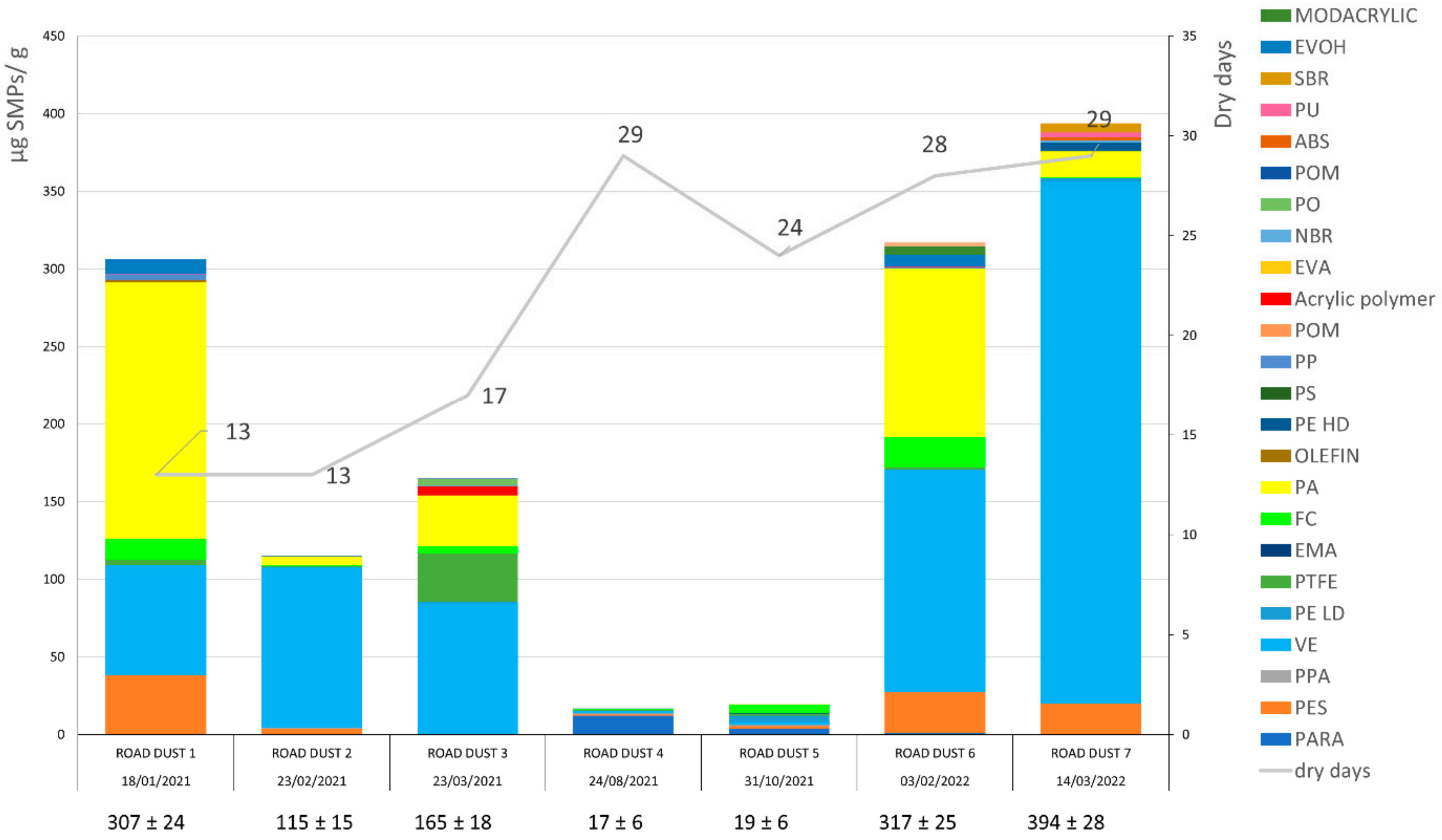

Fig. S2 Weight of SMPs(µg/g) in the HWRD samples analyze

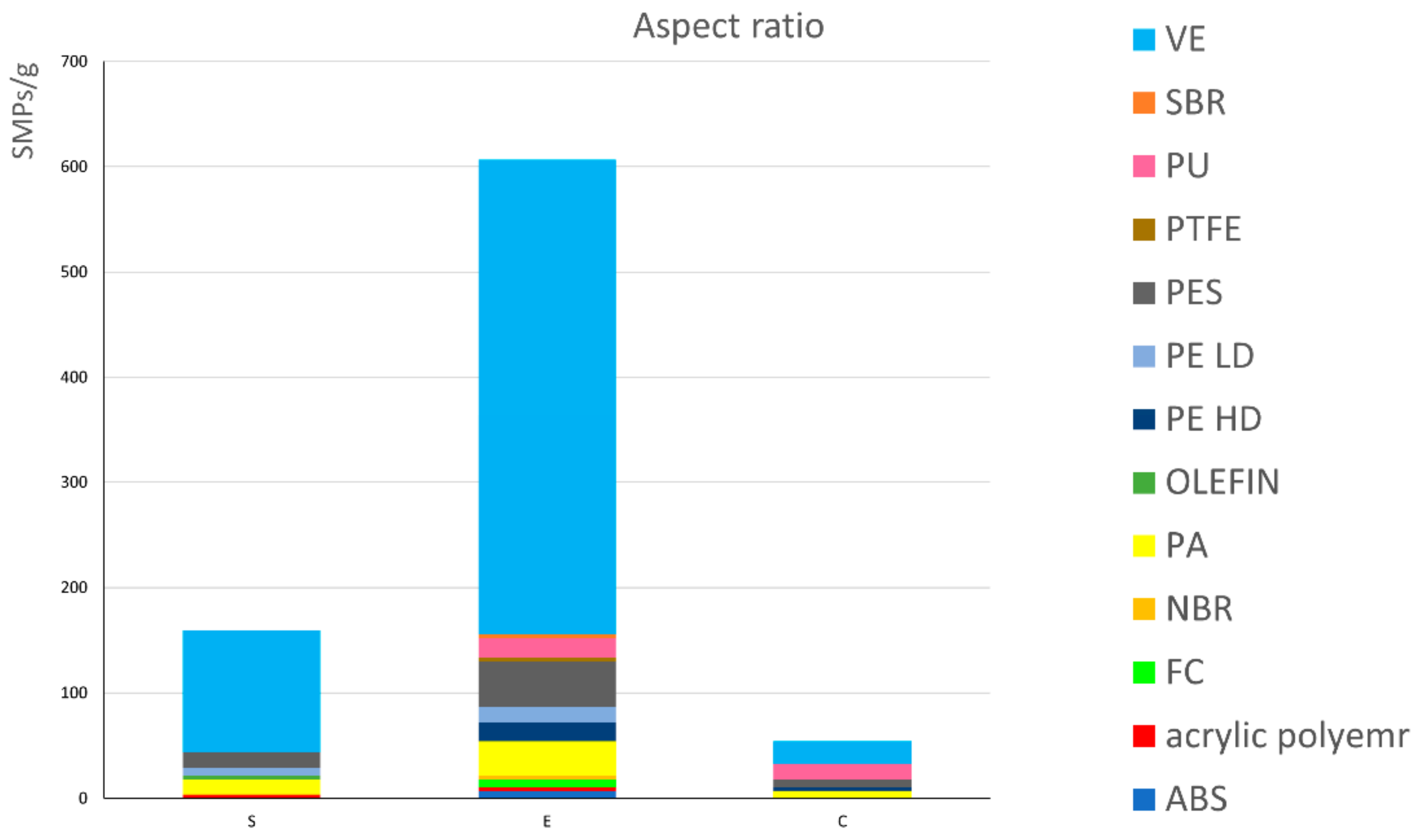

Fig. S3 Ellipsoid (extrapolated from AR) being the most common shape for SMPs in HWRD. When AR =1, particles are considered spherical (S), while when AR =2, particles are elongated/ellipsoidal (E), and they are considered elliptical. When AR ≥ 3, particles are considered cylindrical (C).

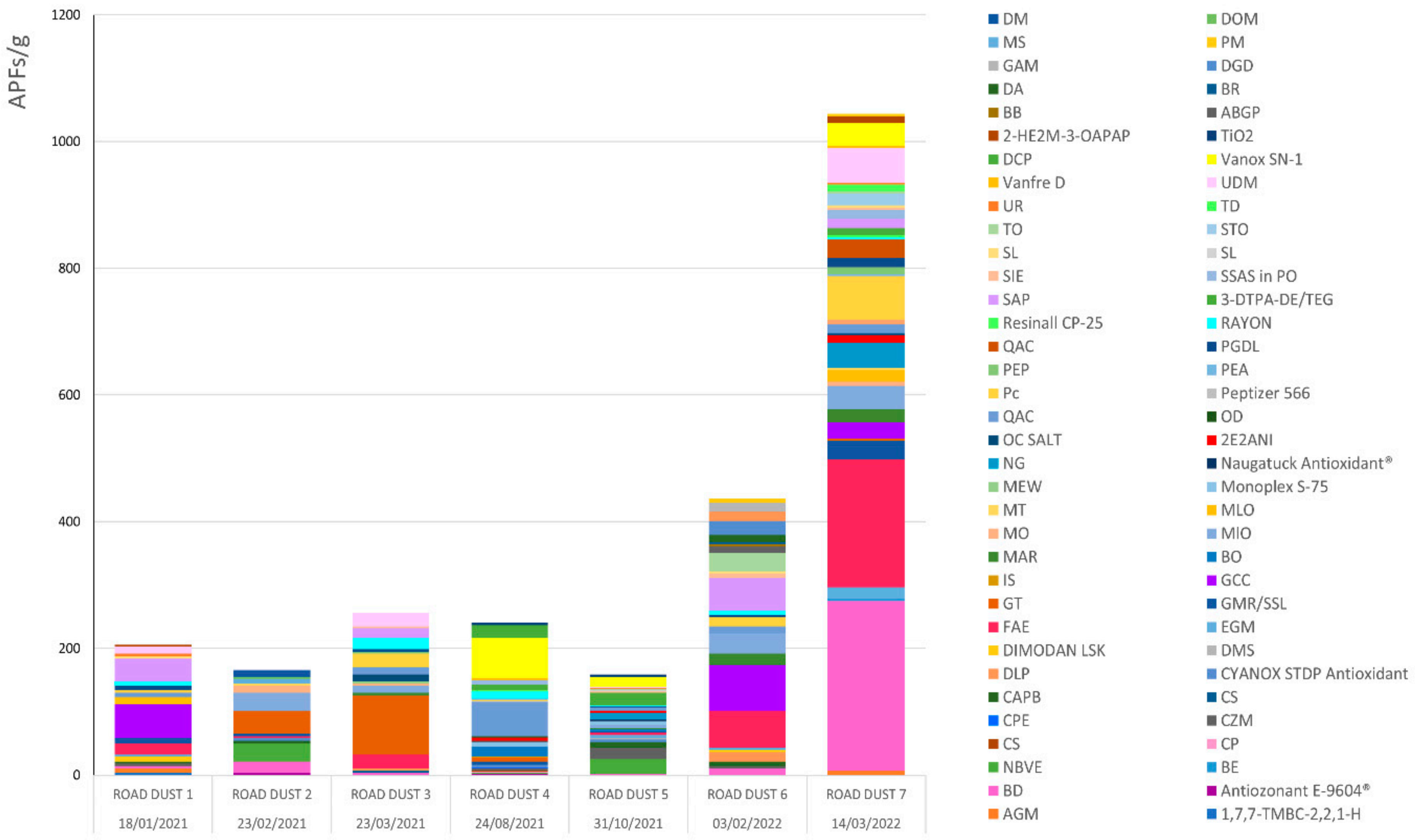

Fig. S4 APFs' abundance (APFS/g) with the list of all singular APFs analyzed in all the HWRD samples

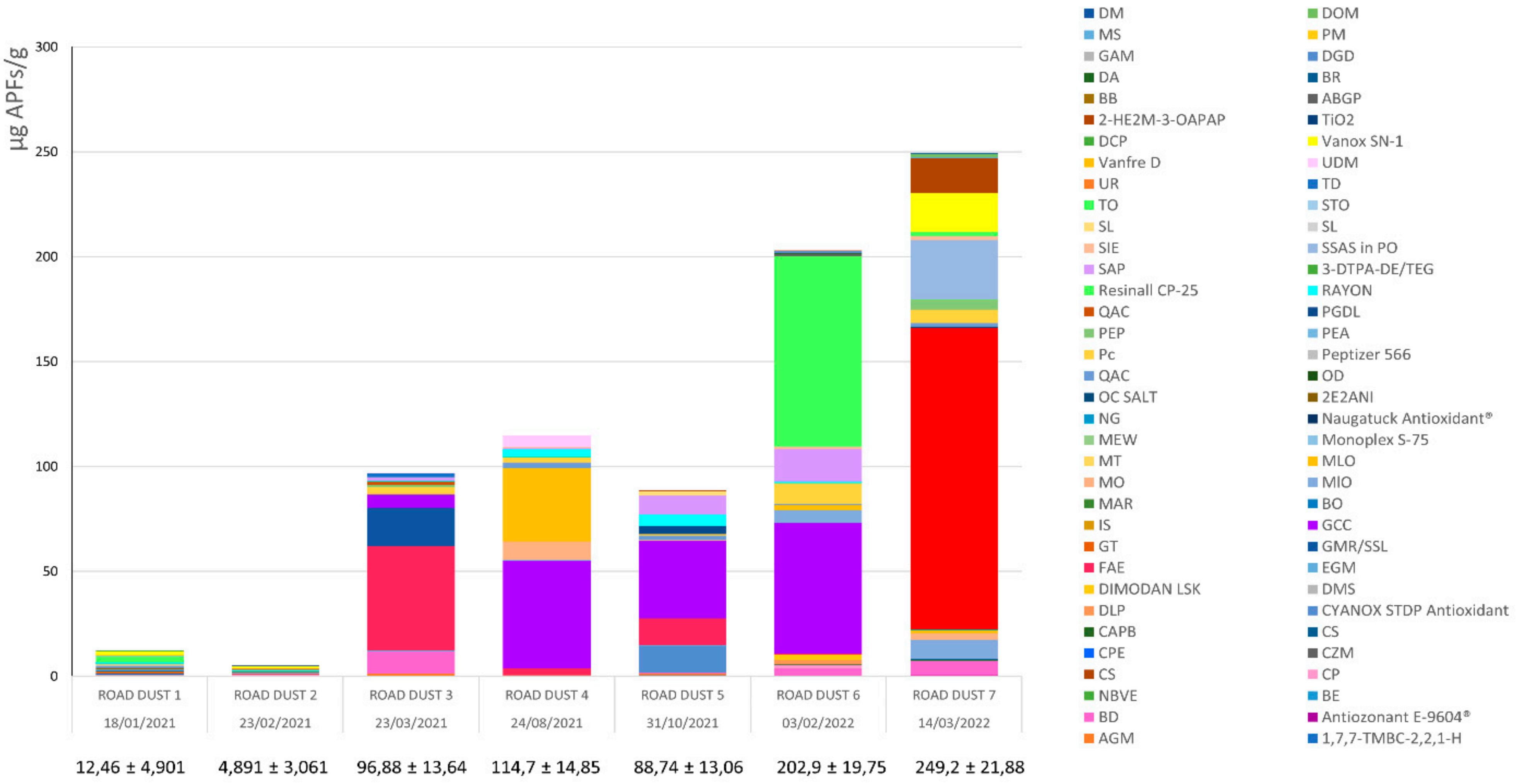

Fig. S5 Weight of APFs (µg/g) in all HWRD samples analyze

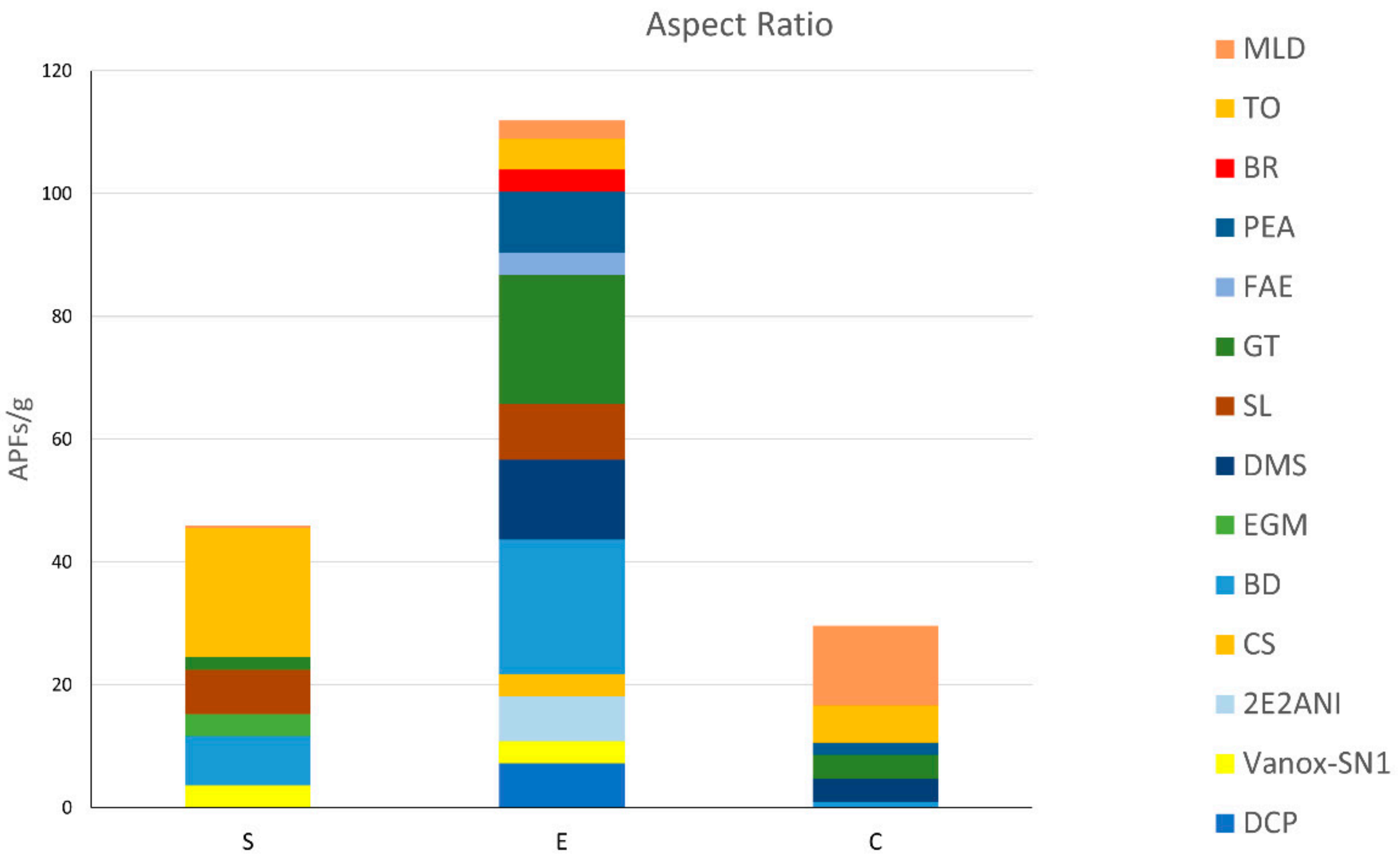

Fig. S6 Ellipsoid (extrapolated from AR) being the most common shape for APFs in HWRD. When AR =1, particles are considered spherical (S), while when AR =2, particles are elongated/ellipsoidal (E), and they are considered elliptical. When AR ≥ 3 particles are considered cylindrical (C).
